# Bilateral Gram‐Negative Facial Abscesses After Polynucleotide Injections

**DOI:** 10.1111/jocd.71060

**Published:** 2026-07-09

**Authors:** Mohammed Shanshal, Radhika Bali, Emily Moon, Rosanna Fox, Sumbel Khan

**Affiliations:** ^1^ Department of Dermatology Imperial College Healthcare NHS Trust London UK; ^2^ Imperial College London London UK

## Abstract

**Background:**

Polynucleotide (PN) injections are increasingly used for facial rejuvenation, but severe infectious complications remain poorly characterized.

**Case Report:**

We report an otherwise healthy 57‐year‐old woman who developed progressive bilateral cheek inflammation after facial PN injections. Initial features resembled expected post‐injection swelling, but worsening pain, fluctuance, rising inflammatory markers, and ultrasound‐confirmed collections prompted aspiration and incision and drainage. Microscopy demonstrated gram‐negative bacilli, and operative cultures grew *Enterobacter cloacae* complex and *Klebsiella variicola* . The patient improved after source control and targeted antimicrobial therapy, but 6‐month follow‐up showed residual post‐drainage atrophic cheek scarring, including a right‐sided soft‐tissue contour depression.

**Conclusion:**

A targeted literature search did not identify a prior published culture‐confirmed gram‐negative abscess after PN injection. This case highlights the need for early imaging, aspiration for culture, and consideration of gram‐negative pathogens when post‐cosmetic‐injection inflammation is progressive, fluctuant, or not responding to usual gram‐positive cover.

## 1 Introduction

Polynucleotide (PN) injections, often marketed as “skin boosters,” are increasingly used for facial rejuvenation and related indications, but high‐quality safety data remain limited [1]. In a recent systematic review of PN use in aesthetic medicine, only nine studies including 219 patients were identified, and reported adverse effects were generally mild and transient [2]. A targeted literature search did not identify a prior published case of culture‐confirmed gram‐negative facial abscess formation after PN injection. By contrast, abscesses are a recognized but uncommon complication of hyaluronic acid filler injections [3] and rare gram‐negative infections after hyaluronic acid fillers have been reported, mainly involving *Serratia marcescens* rather than Enterobacter *or* Klebsiella *species* [4, 5]. In this context, we report bilateral, culture‐confirmed gram‐negative facial abscesses after PN injections—a severe complication that can initially resemble expected post‐injection inflammation.

## 2 Case Report

**FIGURE 1:**
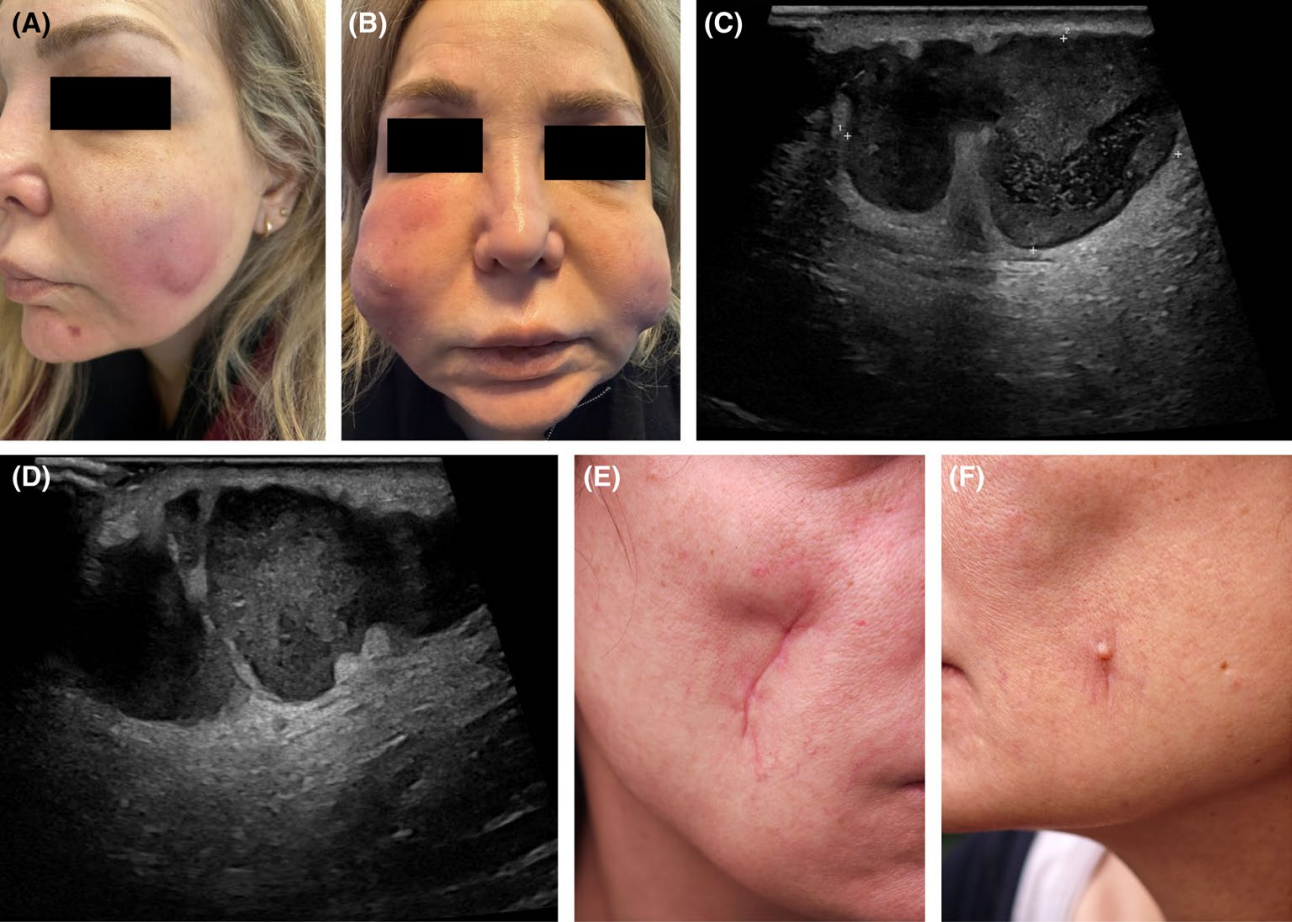
Clinical, ultrasound and post‐drainage course of bilateral facial abscesses. (A) Initial presentation showing erythema and induration of the left cheek. (B) Five days later, bilateral cheek swelling had progressed, with tenderness and fluctuance before surgical intervention. (C, D) Corresponding ultrasound images of the left (C) and right (D) cheeks demonstrating complex, well‐defined subcutaneous fluid collections with internal echoes, consistent with abscess formation. (E) Six‐month follow‐up photograph of the right cheek showing a post‐drainage atrophic scar with tethering and soft‐tissue contour depression. (F) Six‐month follow‐up photograph of the contralateral cheek showing a smaller post‐drainage atrophic scar.

A 57‐year‐old woman, otherwise healthy with no diabetes, immunosuppression, or relevant comorbidity, developed bilateral cheek erythema and oedema within 24 h of receiving a polynucleotide injection (Plenhyage XL Strong; Bioformula s.r.l., Usmate Velate, Italy) in December 2025. On initial presentation, her temperature was 37.9°C, with elevated inflammatory markers (white blood cell count, 12.2 × 10^9^/L; C‐reactive protein, 11.2 mg/L). She received a single intravenous dose of flucloxacillin, reflecting initial empiric treatment for presumed gram‐positive cellulitis, and was discharged with oral antibiotics. Over the next 5 days, symptoms progressed with rising C‐reactive protein (57 mg/L; white blood cell count, 11.1 × 10^9^/L), and she re‐presented to the hospital with enlarging, tender, fluctuant bilateral cheek masses (Figure 1A,B). On post‐injection day 6, ultrasound demonstrated complex subcutaneous collections (left, 2.8 × 1.2 cm; right, 2.3 × 1.5 cm) with surrounding cellulitis (Figure 1C,D). Aspirate microscopy showed numerous leukocytes and gram‐negative bacilli, which prompted admission for intravenous ceftriaxone and metronidazole and urgent incision and drainage. Intraoperative cultures grew *Enterobacter cloacae* complex and *Klebsiella variicola* ; mycobacterial cultures were negative. Intravenous therapy was transitioned to oral trimethoprim‐sulfamethoxazole and metronidazole, with clinical resolution and normalization of inflammatory markers before discharge. At 6‐month follow‐up, clinical photographs demonstrated bilateral post‐drainage atrophic scars, more severe on the right cheek, where tethering and soft‐tissue volume loss produced a visible facial contour depression (Figure 1E,F).

## 3 Discussion

This report is limited to a single patient and cannot establish causality or the source of contamination. No product‐batch testing, environmental sampling, or clinic infection‐control assessment was available. Plausible pathways include product‐related contamination before use, handling‐related contamination after opening, procedural inoculation during injection, or environmental contamination during the treatment episode. The patient had no documented host risk factor, making an exogenous contamination pathway plausible, but the available data cannot distinguish between product‐related, handling‐related, or procedural sources. However, it highlights a diagnostic pitfall: early post‐injection erythema and swelling may resemble expected treatment effects, yet progressive pain, rising inflammatory markers, or fluctuance should prompt imaging and aspiration for microbiological confirmation. In this patient, early microscopy showing gram‐negative bacilli and subsequent cultures necessitated operative source control and targeted antimicrobial therapy. *Enterobacter cloacae* complex and *Klebsiella variicola* are not typical dominant skin commensals [6]. Their recovery from operative cultures is therefore clinically meaningful: it supports an atypical post‐procedural gram‐negative infection rather than routine community‐acquired cellulitis and raises concern for exogenous inoculation or healthcare/environmental contamination. In other injection‐based procedural settings, outbreaks involving Klebsiella and Enterobacter species have been linked to infection‐control lapses, including contamination of single‐use vials that were then used for multiple patients [7]. Although biofilm cannot be inferred from this acute presentation, biofilm is a recognized consideration in late or delayed filler‐related complications, including nodules, chronic inflammatory reactions and abscesses; this reinforces the importance of microbiological sampling and drainage when collections are present [8]. These observations support maintaining a low threshold for culture‐directed management when gram‐negative bacilli are seen on early microscopy after cosmetic injections. Initial empiric gram‐positive cover may be insufficient when post‐injection inflammation is progressive, bilateral, fluctuant, associated with rising inflammatory markers, or fails to improve within the expected early course. The residual cosmetic sequela is also clinically important. In this patient, the right‐cheek depression is most consistent with post‐abscess soft‐tissue loss and scar tethering following abscess formation and drainage. Once infection is fully resolved and scar maturation is adequate, potential corrective options include referral for facial scar and contour revision, with consideration of scar release, autologous fat grafting or other volume‐restoring procedures, and selected resurfacing or vascular laser treatment for residual texture or erythema, depending on scar depth, tethering, patient preference and procedural risk [9, 10].

This severe infectious complication underscores the importance of strengthening oversight for cosmetic injectables and improving post‐marketing surveillance. In England, the Department of Health and Social Care has published its consultation response outlining implementation of a licensing scheme for non‐surgical cosmetic procedures [11]. Suspected adverse incidents should also be reported through established national pharmacovigilance pathways (e.g., the MHRA Yellow Card scheme in the UK) to support signal detection and timely regulatory action [12]. Clinically, progressive pain, rising inflammatory markers, or fluctuance after cosmetic injection should prompt early imaging and aspiration for culture; gram‐negative pathogens should be considered when the course is atypical or not responding to usual gram‐positive cover, and confirmed abscesses require timely source control with culture‐directed antimicrobial therapy. To our knowledge, this is among the first reports of culture‐confirmed gram‐negative abscess formation following PN injections, highlighting a potential shift in the microbiological spectrum of post‐procedural infections.

## Funding

The authors have nothing to report.

## Consent

Written informed consent for publication was obtained.

## Conflicts of Interest

The authors declare no conflicts of interest.

## Data Availability

The authors have nothing to report.
